# GCRP: Integrated Global Chicken Reference Panel from 11,951 Chicken Genomes

**DOI:** 10.1093/gpbjnl/qzaf032

**Published:** 2025-04-15

**Authors:** Di Zhu, Yuzhan Wang, Hao Qu, Chungang Feng, Hui Zhang, Zheya Sheng, Yunliang Jiang, Qinghua Nie, Suqiao Chu, Dingming Shu, Ziqin Jiang, Dexiang Zhang, Lingzhao Fang, Hui Li, Zhenqiang Xu, Yiqiang Zhao, Yuzhe Wang, Xiaoxiang Hu

**Affiliations:** State Key Laboratory of Animal Biotech Breeding, College of Biological Sciences, China Agricultural University, Beijing 100193, China; State Key Laboratory of Animal Biotech Breeding, College of Biological Sciences, China Agricultural University, Beijing 100193, China; National Research Facility for Phenotypic and Genotypic Analysis of Model Animals (Beijing), China Agricultural University, Beijing 100193, China; State Key Laboratory of Swine and Poultry Breeding Industry, Guangdong Key Laboratory of Animal Breeding and Nutrition, Institute of Animal Science, Guangdong Academy of Agricultural Sciences, Guangzhou 510640, China; College of Animal Science and Technology, Nanjing Agricultural University, Nanjing 210095, China; College of Animal Science and Technology, Northeast Agricultural University, Harbin 150030, China; Key Laboratory of Agricultural Animal Genetics, Breeding and Reproduction of Ministry of Education, College of Animal Science and Technology, Huazhong Agricultural University, Wuhan 430070, China; Shandong Provincial Key Laboratory of Animal Biotechnology and Disease Control and Prevention, Shandong Agricultural University, Taian 271018, China; Department of Animal Genetics, Breeding and Reproduction, College of Animal Science, South China Agricultural University, Guangzhou 510642, China; Shijiazhuang Animal Husbandry Technology Promotion Station, Shijiazhuang 050000, China; State Key Laboratory of Swine and Poultry Breeding Industry, Guangdong Key Laboratory of Animal Breeding and Nutrition, Institute of Animal Science, Guangdong Academy of Agricultural Sciences, Guangzhou 510640, China; Wen’s Nanfang Poultry Breeding Co., Ltd., Yunfu 527400, China; Wen’s Nanfang Poultry Breeding Co., Ltd., Yunfu 527400, China; Center for Quantitative Genetics and Genomics, Aarhus University, 8000 Aarhus, Denmark; College of Animal Science and Technology, Northeast Agricultural University, Harbin 150030, China; Wen’s Nanfang Poultry Breeding Co., Ltd., Yunfu 527400, China; State Key Laboratory of Animal Biotech Breeding, College of Biological Sciences, China Agricultural University, Beijing 100193, China; State Key Laboratory of Animal Biotech Breeding, College of Biological Sciences, China Agricultural University, Beijing 100193, China; National Research Facility for Phenotypic and Genotypic Analysis of Model Animals (Beijing), China Agricultural University, Beijing 100193, China; State Key Laboratory of Animal Biotech Breeding, College of Biological Sciences, China Agricultural University, Beijing 100193, China; National Research Facility for Phenotypic and Genotypic Analysis of Model Animals (Beijing), China Agricultural University, Beijing 100193, China

**Keywords:** Chicken, Genotype imputation, Reference panel, Website service, Genome-wide association study

## Abstract

Chickens are a crucial source of protein for humans and a popular model animal for bird research. Despite the emergence of imputation as a reliable genotyping strategy for large populations, the lack of a high-quality chicken reference panel has hindered progress in chicken genome research. To address this, here we introduce the first phase of the 100K Global Chicken Reference Panel (100K GCRP). Currently, two panels are available: a comprehensive mix panel (CMP) for domestication diversity research and a commercial breed panel (CBP) for breeding broilers specifically. Evaluation of genotype imputation quality showed that CMP had the highest imputation accuracy compared to imputation using existing chicken panels in Animal-SNPAtlas and Animal Genotype Imputation Database (AGIDB), whereas CBP performed stably in the imputation of commercial populations. Additionally, we found that genome-wide association studies using GCRP-imputed data, whether on simulated or real phenotypes, exhibited greater statistical power. In conclusion, our study indicates that the GCRP effectively fills the gap in high-quality reference panels for chickens, providing an effective imputation platform for future genetic and breeding research. The project includes 11,951 samples and provides services for various applications on its website at http://farmrefpanel.com/GCRP/#/.

## Graphical abstract

**Figure:**
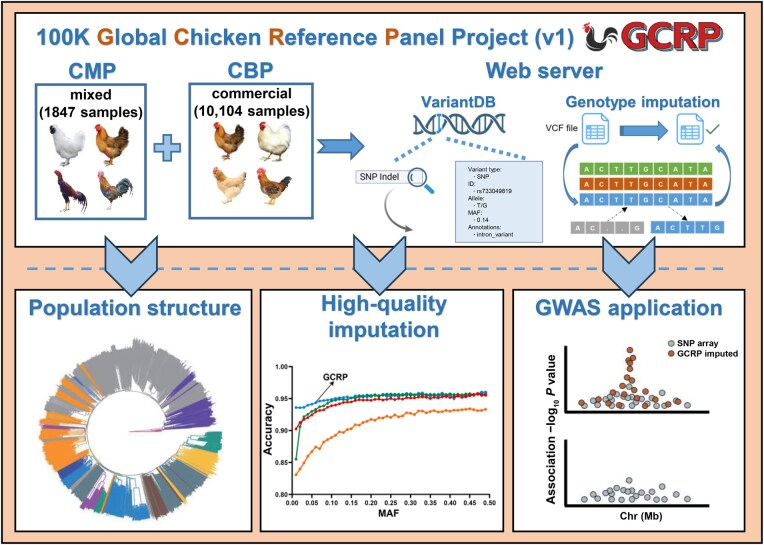

## Introduction

Chickens are the most abundant bird in the world and the most common source of meat for humans. They were domesticated approximately 8000 years ago from red jungle fowl (*Gallus gallus*), endemic to tropical South and Southeast Asia [1]. The spread of domesticated chickens from Asia to the rest of the world coincided with the establishment of trade routes [2]. Currently, the subset of domesticated chickens for human consumption (known as broilers) are so prevalent that they represent how human diets have altered the biosphere. For example, in the early 1950s, the Chicken-of-Tomorrow Program was implemented, significantly increasing broiler growth rates and causing up to a five-fold increase in individual biomass [3]. Chicken thus has the potential to be a biostratigraphic marker species of the Anthropocene [4].

The red jungle fowl was the first bird and among the first vertebrates to have its genome sequenced [5]. The public catalogue of variant sites [Single Nucleotide Polymorphism Database (dbSNP), v106] contains approximately 23.43 million (M) single nucleotide polymorphisms (SNPs) and 2.40 M short insertions and deletions (indels). These resources have greatly contributed to gene discovery in genome-wide association studies (GWAS) [6]. Furthermore, thousands of breeding individuals have now been successfully sequenced through advancements in low-coverage sequencing (LCS) strategies [7–9], allowing for the identification of gaps in SNP arrays that only include fewer SNPs and facilitating the feature optimization of genomic prediction [10–12].

A high-quality reference panel enhances the accuracy of genotype imputation across populations and is thus essential for genomics research. Through the 1000 Bull Genomes Project, the cattle industry has published thousands of sequencing datasets that have been applied to GWAS and genomic prediction [13]. For example, two candidate genes (*RASGRP3* and *GDF-3*), which are potentially associated with intramuscular fat in pigs, were newly identified following genotype imputation based on a high-quality porcine haplotype reference panel [14]. A similar panel would likewise be extremely useful for furthering genetic studies on domesticated chickens. Currently, the most comprehensive panel available is the Chicken2K project (http://chicken.ynau.edu.cn/index/about/index.html); however, it primarily focuses on genomic diversity within wild jungle fowl and domesticated populations, without addressing the breeding of commercial broilers. Furthermore, although several multispecies databases include chickens [15–17], the limited amount of species-specific reference haplotypes has hindered accurate genome research in the world’s most popular poultry. Hence, we formulated the 100K Global Chicken Reference Panel (100K GCRP) project. This endeavor aims to discover, genotype, and provide accurate haplotype data for domesticated chickens worldwide, including commercial broiler breeds.

In this study, we present the results from the first phase of our project. Here, our aim was to compare different strategies for reference panel construction, as well as their applications in genotype imputation and causal gene fine-mapping studies. We conducted LCS of 10,104 broiler individuals for commercial breed panel (CBP), and deep sequencing of 1847 domesticated individuals for comprehensive mix panel (CMP). Our findings offer insight into chicken genetic variation that is more comprehensive and consistent than those of previous studies. The 100K GCRP will enable us to better understand the landscape of functional variation, genetic associations, and artificial selection in chickens.

## Database content and computation methods

### Sample collection and sequencing

The CBP consisted of 11,340 individuals from five populations, including four yellow-feather broiler lines (YB1, YB2, YB3, and YB4) and one white-feather broiler line (WB1). After removing duplicate or highly related samples (as mentioned below), the sample sizes were 2545, 946, 1968, 2760, and 1885, respectively, leaving a total of 10,104 samples. All subjects originated from commercial breeding companies and were subjected to LCS at depths ranging from 0.4× to 1.7×. Genomic DNA was extracted from blood samples using a DNeasy Blood & Tissue Kit (Catalog No. 69504, QIAGEN, Hilden, Germany). Extracted DNA quality was assessed in a NanoDrop spectrophotometer and verified with 1% agarose gel electrophoresis. Samples were quantified using a Qubit 2.0 Fluorimeter, and then diluted to 40 ng/ml in 96-well plates. A Tn5-based library generation method was employed to produce sequence libraries, as described previously [7]. The final libraries were sequenced using the MGISEQ-2000 platform, generating paired-end reads of 2 × 100 bp.

The CMP used data downloaded from the National Center for Biotechnology Information (NCBI) Sequence Read Archive (SRA), including 1847 re-sequenced samples across 114 breeds/lines. The samples encompassed both commercial broiler and layer chicken populations, as well as local breeds worldwide (Table S1).

Both CBP and CMP are freely available to the public without registration or login requirements (http://farmrefpanel.com/GCRP/#/).

### Genotyping and haplotype construction

Raw sequencing reads for LCS samples were trimmed using Trimmomatic (v0.36) [18] and subsequently mapped to the GRCg6a reference genome (NCBI RefSeq: GCF_000002315.6) using the GTX-One platform [19]. GTX-One is an Field Programmable Gate Array (FPGA)-based hardware accelerator optimized for Burrows-Wheeler-Alignment (BWA) [20] and Genome Analysis Toolkit (GATK) [21] best-practice workflows, including marking polymerase chain reaction (PCR) duplicates and indexing BAM files. Quality checks were performed using indel realignment and base quality score recalibration (BQSR) modules within GATK. Next, SNP variants and allele frequency were estimated in BaseVar (v1.01) [22]. After filtering out SNPs with population depth < 1.5× interquartile range (IQR) and > 2 allele types, 42.23 M candidate SNPs were retained for imputation. Genetalks (China) conducted SNP imputation, using accelerated Sequencing To Imputation Through Constructing Haplotypes (STITCH) [23] software. For parameter *K* (the number of ancestral haplotypes), we conducted a gradient test ranging from 5 to 40 and compared genotype consistency (GC) with 64 high-depth sequencing samples (Figure S1). The results showed that accuracy increased most significantly between *K* = 5 and *K* = 10, while changes in accuracy were smaller when *K* exceeded 10. However, computation time increased progressively with higher *K* values. Balancing accuracy and computational resource consumption, we set *K* to 20. Post-imputation, SNPs with minor allele count (MAC) < 5, imputation information (INFO) score < 0.40, and missing rate > 0.80 were excluded, retaining 20.41 M variants. To remove potentially duplicate or highly related samples, we used PLINK [24] with the “--genome” option to calculate the identity by descent (IBD) between individuals. If the “PI_HAT” value between two samples was > 0.85, one of the samples was removed. To validate the accuracy of genotypes in the CBP panel, a total of 64 samples were randomly selected from YB1 and subjected to high-depth sequencing, achieving an average sequencing depth of 58.79×, to serve as the validation set.

For the CMP, we downloaded SRA files for each individual and converted them to Fast Quality (FASTQ) files using SRA Toolkit (v3.0.2). Similar to the CBP, all FASTQ files were processed to generate Genomic Variant Call Format (gVCF) files based on the GRCg6a reference genome, and then joint calling was executed for all CMP individuals using the GTX-One platform. Subsequently, SNPs were filtered using GATK (v4.1.9.0) VariantFiltration with the parameters “QD < 2.0, MQ < 40.0, FS > 60.0, SOR > 3.0, MQRankSum < −12.5, ReadPosRankSum < −8.0, QUAL < 30”. Indels were filtered using the parameters “QD < 2.0, FS > 200.0, ReadPosRankSum < −20.0, QUAL < 30”. After quality control, 39.33 M SNPs and 4.79 M indels were retained.

Both CBP and CMP variants were phased using Beagle5.2 [25] to generate haplotypes for all samples. The polymorphic spectrum and potential effects of each SNP are accessible via the “VariantDB” function on the GCRP website. Reference panels can also be obtained from the “Download” page for localized analysis.

### Population genetic analyses

Population genetic analyses were performed to understand the genetic structure of breeds and to provide data on CMP and CBP genetic backgrounds for genotype imputation. First, phylogenetic relationships of CMP samples were evaluated, and principal component analysis (PCA) was performed to determine any clustering. Samples (*n* = 1847) were divided into 12 sub-populations based on origin. The identical-by-state (IBS) distance matrix within CMP samples was calculated using PLINK (v1.9) [24] with the parameters “--genome” and “--distance-matrix”. This matrix was the foundation for constructing an unrooted neighbor-joining tree in FastME [26]. Finally, PCA for the CMP was executed using SMARTPCA [27] based on linkage disequilibrium (LD)-pruned data derived from PLINK (parameters: --indep-pairwise 50 10 0.2).

The CBP contained populations with a larger sample size and met broader criteria for genetic analysis. Variants and haplotypes shared among the five groups were investigated. To lower the possible impact of genotyping errors on each population, only SNPs with MAC > 3 were considered polymorphic variants in each group. An analysis of SNPs, haplotype sharing, and polymorphisms was conducted across populations. Samples in the CBP were then combined with three CMP populations that had sample sizes > 100, including red jungle fowl, Indian subcontinent natives (ISN), and chicken from Xizang Autonomous Region of China (XZC). To quantify nucleic acid diversity, “pi” [28] was computed using VCFtools (v0.1.16) [29] (parameters: --pi-window 300 k --step 100 k) with SNPs shared between CBP and CMP.

For haplotype diversity analysis, overlapping SNP density was further reduced via selecting one SNP from every 20-SNP window and ensuring that SNPs chosen from neighboring windows were approximately equidistant. The analysis utilized 428,000 SNPs, and a sliding window-based approach was employed to construct haplotypes, Window size was set to 8 SNPs, with no overlap between consecutive windows. For each block, haplotype diversity (*H*) per population was calculated, as described previously [30]:


(1) $$H=\frac{N}{N-1}(1-\sum_{i}x_{i}^{2})$$

where *N* is the sample size and *x_i_* is the frequency of haplotype *i* within populations.

### Imputation assessment

Assessment of imputation effectiveness used 205 CMP samples with > 20× sequencing depth as test data (Table S2). We used their genotypes obtained from GATK best-practice as the gold standard, and later excluded these samples from the CMP for further evaluation. To assess whether GCRP had an advantage in genotype imputation over other panels, chicken panels downloaded from the Animal-SNPAtlas database [15] and the Animal Genotype Imputation Database (AGIDB) [17] were used as controls.

Considering real imputation practical, two scenarios were tested. The first aimed to assess imputation from low- or high-density arrays to whole-genome sequencing (WGS). Gold-standard GATK results were downsampled using Affymetrix 600K and Illumina 60K SNP array datasets. Subsequently, imputation was performed using Animal-SNPAtlas, AGIDB, CBP, and CMP panels across Beagle5.2, IMPUTE5 (v1.1.5) [31], and Minimac3 (v2.0.1) [32]. The second scenario was designed for LCS. Sequencing data at 1× depth were randomly extracted from the original FASTQ files for each test sample and subjected to imputation using GLIMPSE (v1.1.1) [33] and QUILT (v1.0.3) [34].

Accuracy was assessed via computing GC for each individual, defined as number of correctly imputed genotypes divided by total number of imputed genotypes. Prior to GC computation, definitive genotypes were assigned to loci based on the highest posterior probability provided by imputation software. Furthermore, allelic dosage *r*^2^ was calculated for every imputed SNP locus (*r*^2^ = square of correlation coefficient between imputed allelic dosage and true dosage). All evaluations of genotype imputation accuracy were performed on the medium-sized chromosome 6 (Chr6) to minimize computational complexity.

### Application of GCRP in GWAS

An additional commercial population was used for assessing the utility of GCRP in GWAS. The additional population comprised 4749 samples from the same generation, all subjected to LCS. Genotype imputation was performed with the CBP and GLIMPSE. After imputation, loci were filtered using minor allele frequency (MAF) > 0.01 and INFO score > 0.4, leaving 9.5 M SNPs for GWAS.

Both simulated phenotypes and real anonymized phenotype A were used in GWAS. Simulated phenotypes were generated from real genotypes in R package SIMER (https://github.com/xiaolei-lab/SIMER), based only on additive genetic and residual effects. Heritability was set at 0.3, with 100 causal mutations randomly selected from all included SNPs. Their effects were sampled from the default normal distribution of SIMER. Residual terms were sampled from another normal distribution with variance adjusted to heritability.

GWAS was performed using the mixed linear model implemented in the Genome-wide Complex Trait Analysis (GCTA; v1.92.0) with the “mlma” option [35]. The model was specified as:


(2) y=Xβ+αb+Wg+e  

where **y** is a vector of phenotypes; for the real trait, **β** is a vector of fixed effects, including batch and chicken coop effects; for the simulated trait, **β** only includes the overall mean; **b** is a vector of genotypes (coded 0, 1, and 2); **α** is the effect of the test SNP; **g** is a vector containing random polygenic effects and **g** ∼ *N* (0, **G**σ^2^_g_), where **G** is the genomic relationship matrix (GRM) constructed using LCS SNPs and σ^2^_g_ is variance explained by SNPs; **e** is a vector of random residual effects and **e** ∼ *N* (0, **I**σ^2^_e_), where **I** is the identity matrix; and **X** and **W** are design matrices connecting phenotypes to fixed effects and random polygenic effects, respectively, where **X** is the identity matrix of the simulated trait.

## Implementation and results

### GCRP

**Figure 1:**
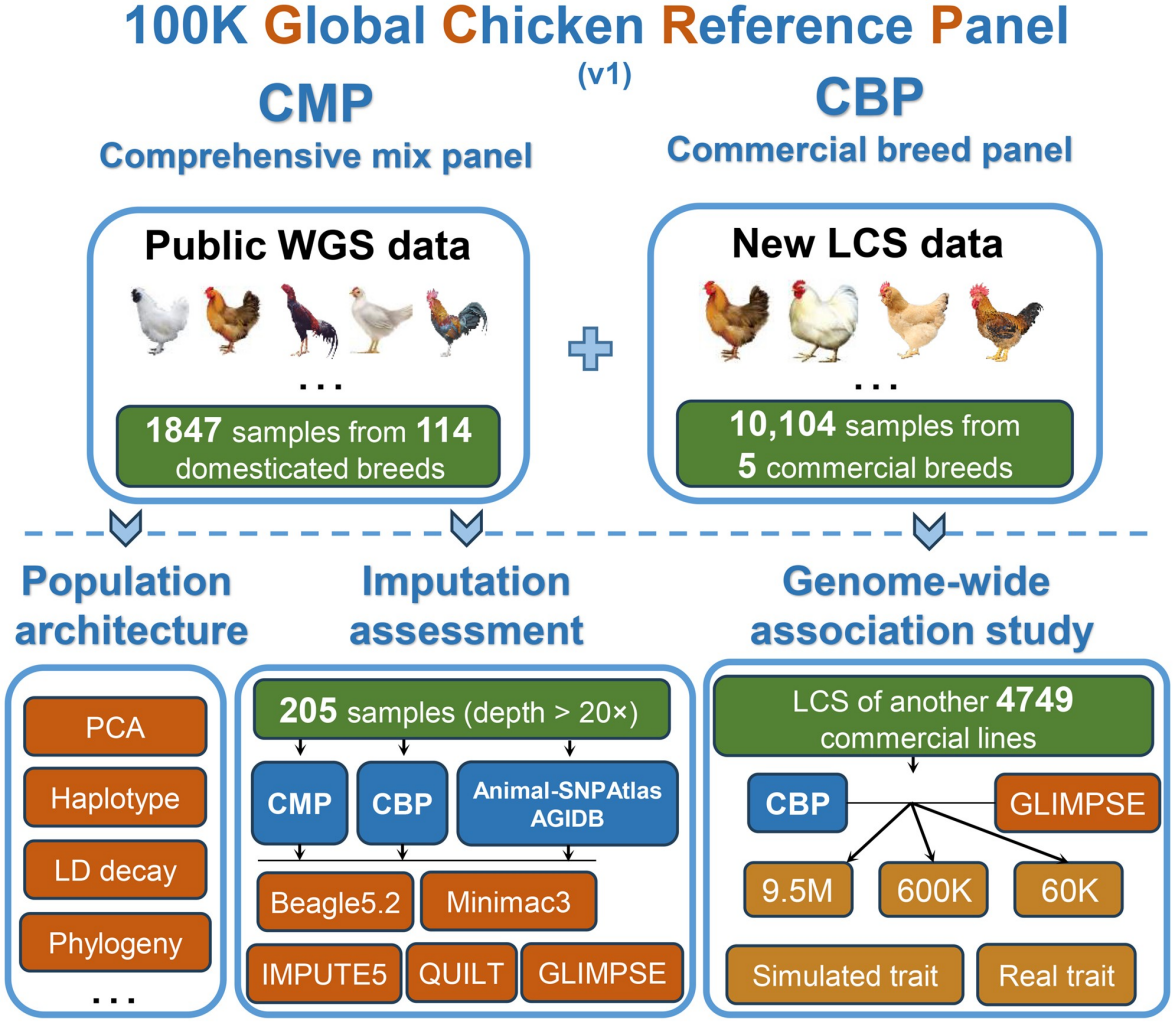
Flow chart of the GCRP database and study design CMP is a domestication panel for thousands of chicken breeds and CBP is an improvement panel for tens of billion commercial chickens. “9.5M” refers to the 9.5-million-SNP set generated in this study; “600K” and “60K” represent the Affymetrix 600K and Illumina 60K SNP arrays, respectively. CMP, comprehensive mix panel; CBP, commercial breed panel; WGS, whole-genome sequencing; LCS, low-coverage sequencing; GCRP, Global Chicken Reference Panel; LD, linkage disequilibrium; PCA, principal component analysis; AGIDB, Animal Genotype Imputation Database.

We generated the CMP using 1847 individuals from 114 breeds spanning various continents (Table S1). Additionally, we generated a CBP specifically for chicken breeding, encompassing 10,104 individuals from 5 major commercial breeds (Table S3). The two panels were combined to form the pilot phase of the GCRP database (**Figure 1**). Overall, we identified 48.02 M SNPs and 4.79 M indels in GCRP, including 26.36 M rare SNPs (0.0001 < MAF < 0.01) and 3.53 M rare indels (0.0001 < MAF < 0.01). Notably, while 66.40% of SNPs in dbSNP were present in GCRP, we discovered 32.47 M novel SNPs, expanding the chicken dbSNP by 139% (Figure S2A). Moreover, 42.59% of SNPs (8.69 M) in CBP did not overlap with those in CMP, indicating significant changes resulting from artificial selection. Only a small portion (5.42%) of indels in CMP overlapped with those in dbSNP (Figure S2B). It is worth noting that CBP temporarily excluded indels due to the limitations of LCS. Figure S2C and D presents the MAF distribution of variants in both panels, showing a higher proportion of rare variants (MAF < 0.05) in the CMP panel. This may be due to the reduced detection capability of LCS for rare variants. To further assess the reliability of genotypes in the CBP panel, we collected 64 high-depth sequencing samples from YB1 as a gold standard to compare with the genotypes obtained via LCS. The results showed an average genotype concordance rate of 0.995 across the 64 validation samples (Figure S2E). Additionally, we calculated the dosage *r*^2^ for imputed variants, dividing them into 1% MAF windows. Although the accuracy decreased in lower-frequency windows, the average dosage *r*^2^ reached 0.913 (Figure S2F), demonstrating the accuracy of the genotypes in the CBP panel.

### Population structure analyses

**Figure 2:**
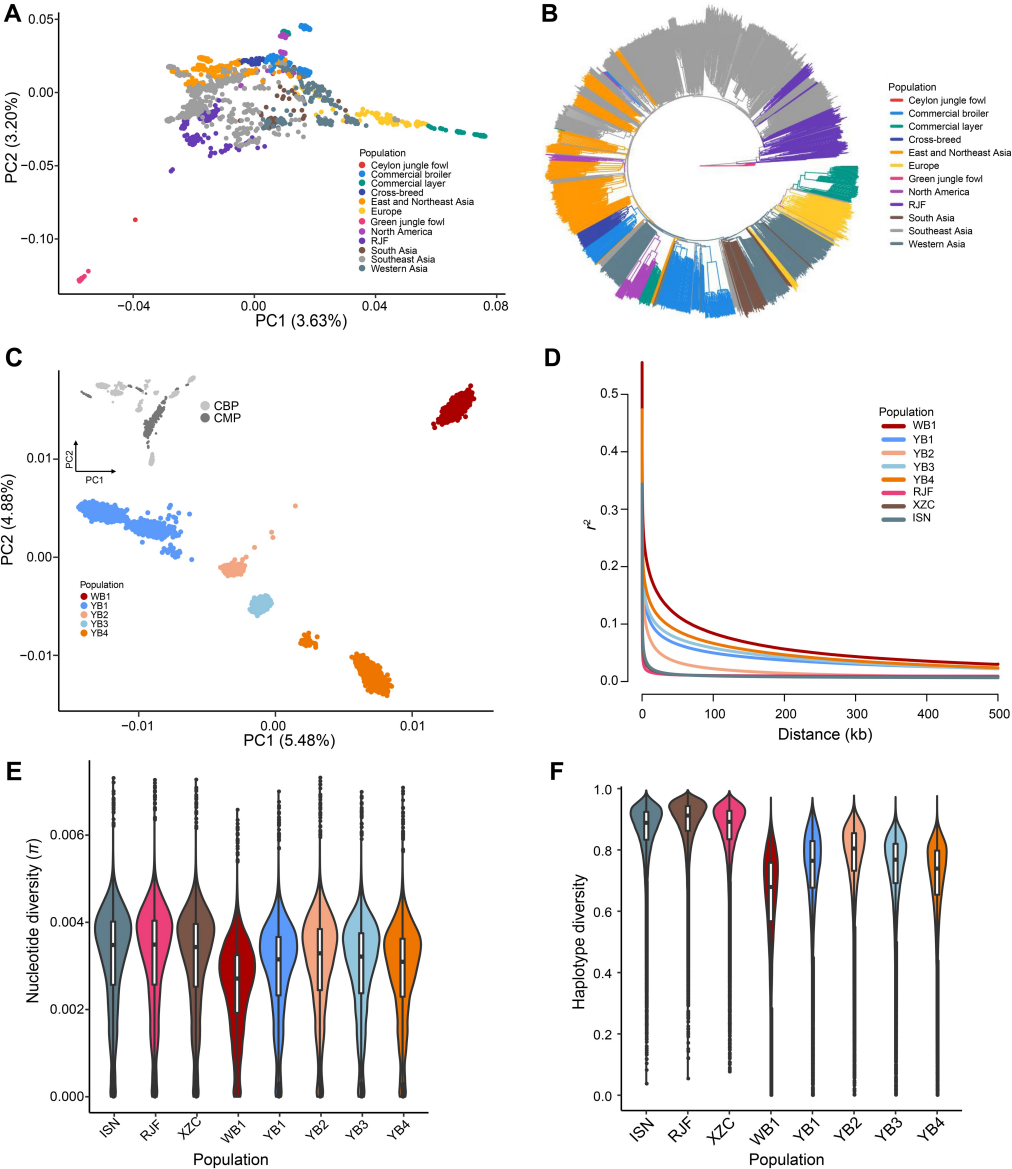
Population genetic analyses of GCRP samples **A**. PCA for CMP. The 12 subgroups are represented by different colors. **B**. Phylogenetic analysis of CMP. The 12 subgroups are indicated by different colors. **C**. PCA for CBP. The relationship between CBP and CMP is illustrated at the top-left corner through overlapping loci in two panels. **D**. LD decay after merging CBP with the three largest CMP populations. **E**. Violin plot displaying nucleotide diversity (*π*) distribution within 300-kb windows for each population. **F**. Violin plot showing haplotype diversity distribution per population. PC, principal component; WB, white-feather broiler line; YB, yellow-feather broiler line; RJF, red jungle fowl; ISN, Indian subcontinent natives; XZC, chicken from Xizang Autonomous Region of China.

For the CMP, we categorized the 114 populations into 12 origin-based subgroups (Table S1). The results of PCA and phylogenetic analyses revealed that most individuals from the same origin tended to cluster together (**Figure 2**A and B). Overall, the domestication trajectory from red jungle fowl to domesticated and commercial chickens was clearly visible. As the domestication process progressed, the red jungle fowl became more distantly related to the other groups.

The CBP samples segregated clearly according to breed and were significantly distinguishable from domesticated breeds in the CMP (Figure 2C). Most SNPs were shared among all five populations, but only 8.89% of haplotypes were shared across all groups (Figure S3). This pattern indicates that artificial breeding of specific traits shaped the genomes of different broilers. To compare the population structure between commercial and native breeds, we combined the CBP with three populations from the CMP for further analysis. The results revealed that the breeds in the CMP (ISN, XZC, and red jungle fowl) exhibited higher nucleotide polymorphism, greater haplotype diversity, and faster LD decay compared to the commercial populations in the CBP (Figure 2D–F). Within the CBP, the YB2 population had the highest variant and haplotype diversity, whereas the WB1 population had the lowest (Figure 2E and F). Haplotype polymorphism was high, while SNP polymorphism was moderate in YB1. These differences in genome architecture accurately reflected breeding stages: YB1 was in the pure breeding stage following hybridization, YB2 was in the conservation stage, and WB1 was in the purebred breeding stage.

### Assessment of genotype imputation

**Figure 3:**
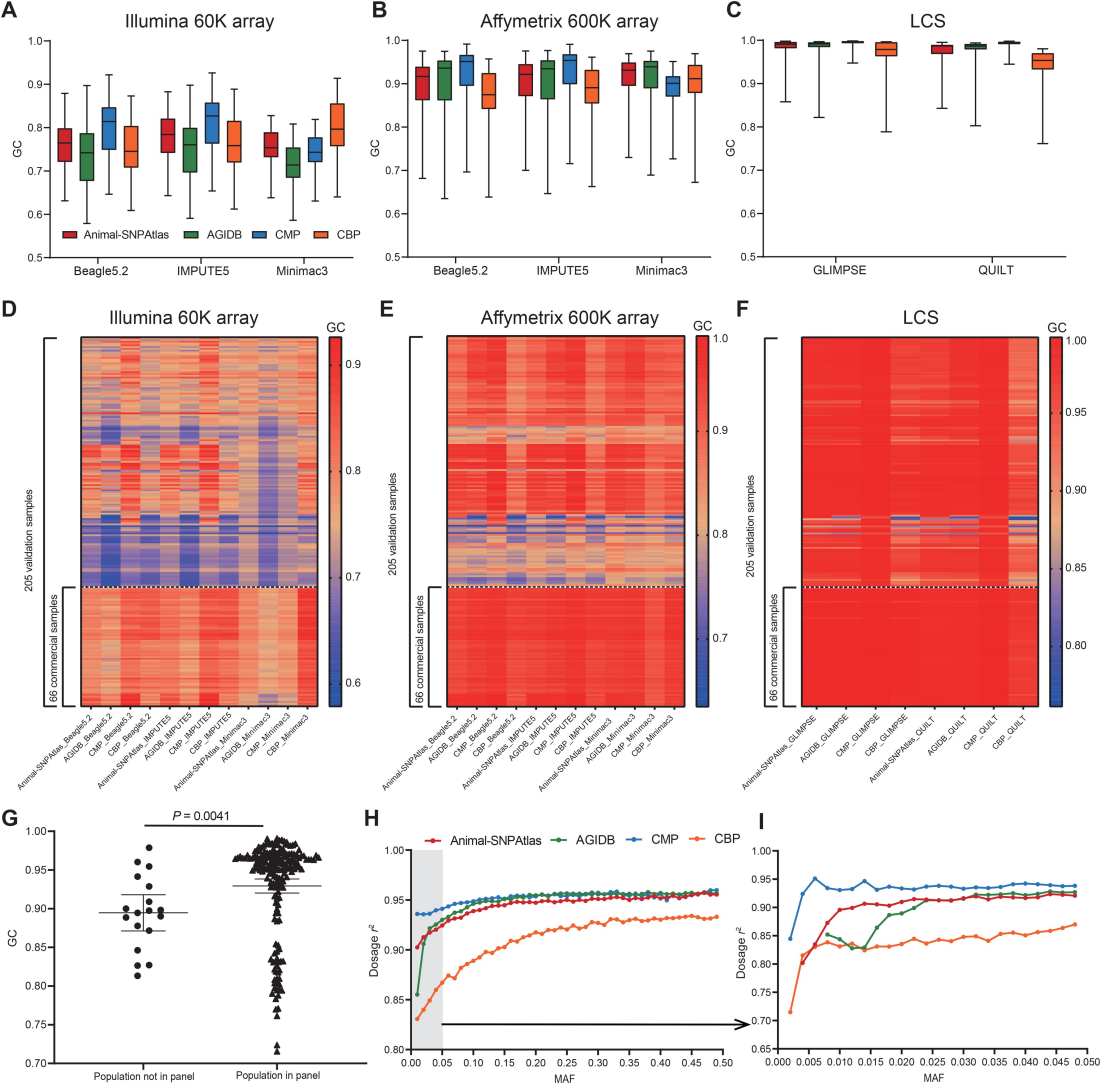
Assessment of genotype imputation accuracy **A**.–**C**. Boxplots showing GC of imputed *vs.* observed genotypes. The assessment was performed using five software (Beagle5.2, IMPUTE5, Minimac3, GLIMPSE, and QUILT) and four reference panels: Illumina 60K array (A), Affymetrix 600K array (B), and LCS (C). Color coding is consistent across (A–C). **D**.–**F**. Heatmaps showing imputed genotype concordance of 205 test samples using Illumina 60K array (D), Affymetrix 600K array (E), and LCS (F). The 66 commercial WPR samples are displayed at the bottom of each heatmap. **G**. Comparison of genotype imputation accuracy between “population in panel” and “population not in panel” samples (*P* = 0.0041, one-sided Student’s *t*-test). **H**. and **I**. Imputation accuracy calculated using GLIMPSE, with MAF binned into intervals of 0.01 (range: 0–0.5) (H) and 0.002 (range: 0–0.05) (I). WPR, white Plymouth rock; MAF, minor allele frequency; GC, genotype consistency.

Our imputation quality assessment showed that sequencing-based strategies had considerably higher GC (**Figure 3**A–C) and dosage *r*^2^ (Figure S4A) than two SNP array-based strategies. Overall, imputation strategies based on LCS demonstrated significantly higher accuracy compared to those based on SNP arrays, though they required more time for computation (Figure S5). For example, the most time-consuming method, QUILT, took 12,540 s, which is 26.7 times longer than the fastest method, Beagle5.2, which took 469 s. The highest GC (0.994) and dosage *r*^2^ (0.961) were obtained using the CMP and QUILT software to impute LCS data. Among array-based imputation results, Affymetrix 600K array performed better than Illumina 60K array across all reference panels and imputation tools. For example, when using CMP as the reference panel, the GC values for three imputation software targeting the Affymetrix 600K array were found to be 0.924, 0.926, and 0.885, respectively. These values were higher compared to the Illumina 60K array, where the GC values were 0.800, 0.810, and 0.748, respectively (Figure 3A and B). Allele dosage *r*^2^ for each estimated SNP aligned with the trends for GC (Figure S4A).

Comparing the GC of the four panels across all 205 validation samples, the results showed that CMP had the highest accuracy in 6 out of 8 imputation scenarios, followed by AGIDB and Animal-SNPAtlas, with CBP having the lowest performance (Figure 3A–C, Figure S4A). This demonstrates that CMP has better imputation performance compared with two published chicken reference panels. Additionally, we found that the accuracy of the test samples is highly correlated with the population background. Since CBP only includes commercial populations, its imputation performance for non-commercial samples is relatively poor. After dividing the 205 test samples into 66 commercial samples and 139 non-commercial samples, we observed that the imputation accuracy achieved using CBP was significantly higher for commercial samples than that for non-commercial samples (Figure 3D–F, Figures S4B, S4C, and S6). For instance, in the case of using Beagle5.2 to impute the Affymetrix 600K array, the average GC for commercial samples using CBP was 0.934, compared to 0.837 for non-commercial samples. For commercial test samples, CBP demonstrated significantly higher imputation accuracy than Animal-SNPAtlas and AGIDB when applied to the low-density Illumina 60K array (Figure S6A). Similarly, in the case of using IMPUTE5 to impute the Illumina 60K array, the average GC achieved by CBP was 0.836, higher than 0.800 and 0.793 obtained by Animal-SNPAtlas and AGIDB, respectively. This may be due to the advantage of a larger sample size in reference panel being more prominent when there is limited known information. These results were consistent with our expectation that CMP and CBP would serve distinct roles in research on domesticated and commercial broiler breeds, respectively. Among all four panels, CMP is the only one that includes small indels. We then evaluated the imputation accuracy of CMP for indels. The results showed that when imputing the low-density Illumina 60K SNP array, the imputation accuracy for indels was higher than SNPs, while it was opposite for the Affymetrix 600K SNP array (Figure S7). Overall, the imputation accuracy for indels was more stable across samples. These results proved the feasibility of using CMP for indel imputation. 

We then divided the 205 samples into two groups, one containing individuals from the same population in the remaining CMP samples (population in the panel) and the other containing no individuals from the same population (population not in the panel). The results demonstrate that the accuracy of related individuals is significantly higher than that of unrelated individuals (one-sided Student’s *t*-test, *P* = 0.0042) (Figure 3G), highlighting the crucial influence of the relationship between the panel and the target sample on imputation accuracy. Furthermore, SNPs with lower MAF tended to have lower dosage *r*^2^ (Figure 3H and I, Figure S8). In the evaluation of rare mutations (0.002 < MAF < 0.05), GCRP successfully detected rare mutations with MAF > 0.005 (Figure 3I).

### Case study of GWAS for chicken complex traits

**Figure 4:**
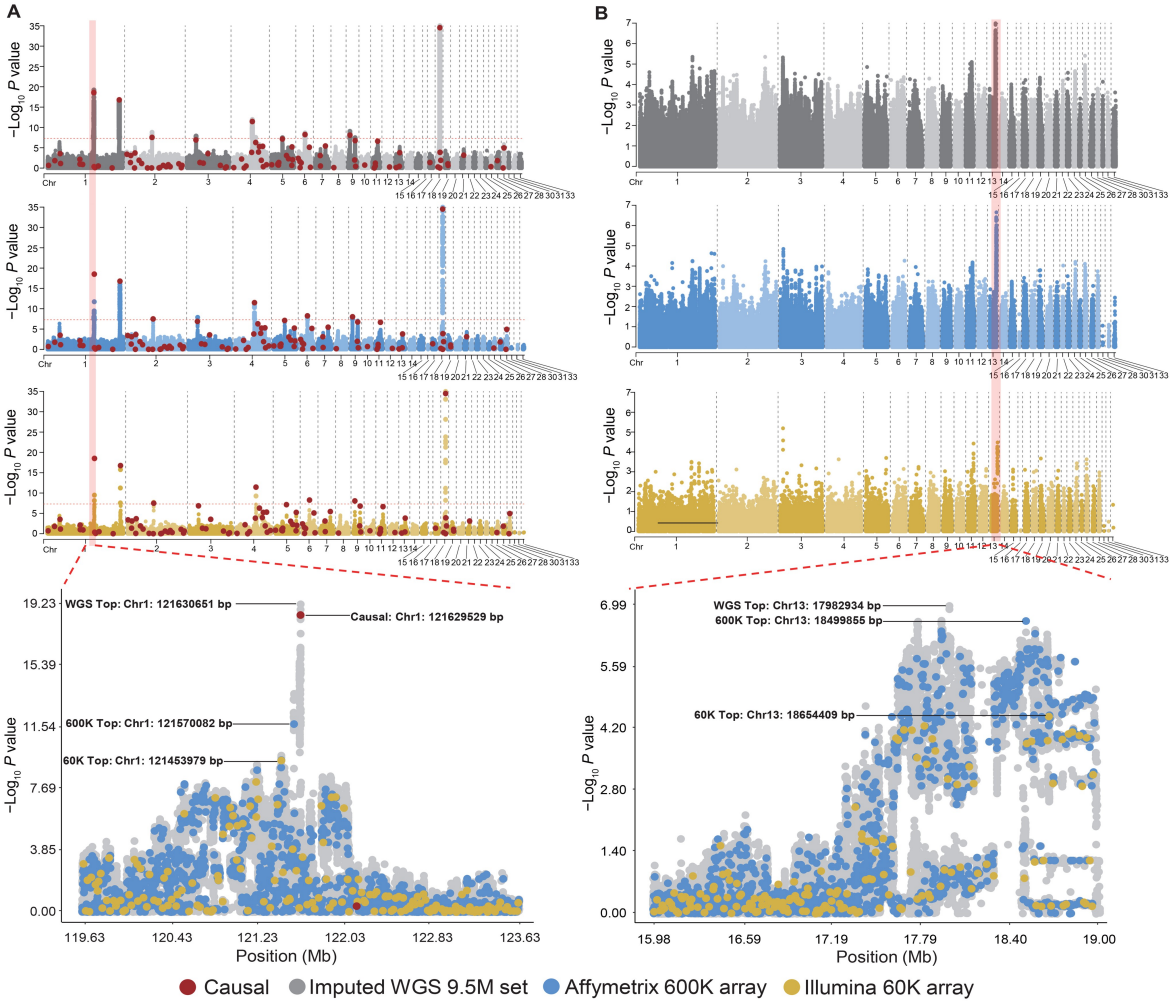
Application of GCRP in GWAS **A**. Manhattan plots and zoomed-in region for simulated trait. **B**. Manhattan plots and zoomed-in region for real anonymized phenotype. The −log_10_  *P* value (Y-axis) is plotted against each SNP position (X-axis). Colors reflect different SNP sets. Red dots indicate locations of true causal mutations in the simulated data. GWAS, genome-wide association study; Chr, chromosome.

Increasing marker density using imputation-based WGS data can enhance the statistical power of GWAS to detect associated signals [36–38]. We employed CBP to perform genotype imputation on an external commercial population of 4749 individuals and obtained a set of 9.5 M SNPs (termed 9.5M set). We then compared three SNP sets (Illumina 60K array, Affymetrix 600K array, and our imputed 9.5M set) in GWAS performance using both simulated and real anonymous phenotypes. Simulated phenotypic heritability was set at 0.3, and we identified seven independent causative mutations exceeding the significance threshold (5 × 10^−8^), representing seven segregating quantitative trait loci (QTLs). All three SNP sets successfully captured the most significant QTL on Chr18 (**Figure 4**A). The Affymetrix 600K array and 9.5M set covered all significant QTLs with false negative rate (FNR) = 0, but the Illumina 60K array missed two QTLs (FNR = 28.57%). Additionally, the 9.5M set revealed that the average distance between the top SNP within each QTL and the causative mutation was 1.2 kb (range: 0–5.3 kb), significantly shorter than that detected by the other two sets (Illumina 60K array: 69.1 kb; Affymetrix 600K array: 75.3 kb).

We observed similar findings in the real phenotype (*h^2^* = 0.24), where the 9.5M set exhibited stronger significance than the two arrays. Only the 9.5M set and the Affymetrix 600K array detected the largest peak on Chr13 (Chr13: 17.41–18.97 Mb) (Figure 4B). Our results substantiate the hypothesis that GCRP provides distinct advantages in GWAS over low- or high-density SNP arrays.

### GCRP web-based services

**Figure 5:**
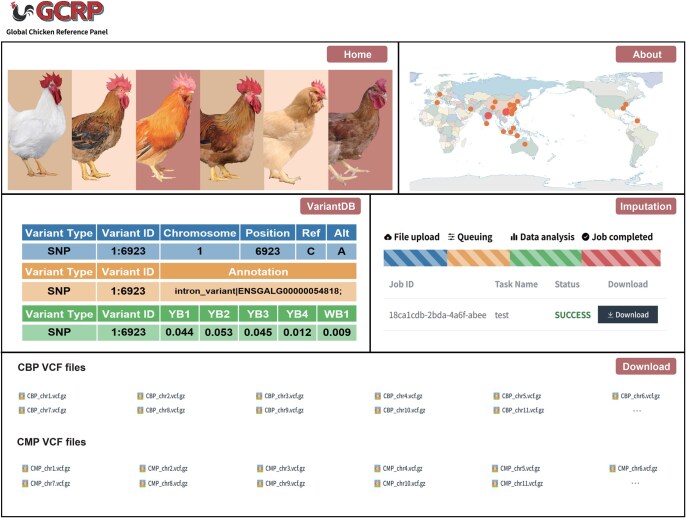
Summary of GCRP web-based services Main modules in GCRP are “Home”, “About”, “VariantDB”, “Imputation”, and “Download”. The “About” page presents the geographical distribution of breeds collected in this study. The base world map is from China Standard Map Service (http://bzdt.ch.mnr.gov.cn/; Approval No. GS(2016)1663). The circle sizes and colors correspond to the sample size for each study area, indicating the number of unique breeds. On the “VariantDB” page, various types of specific information about the recorded variants are displayed, such as allele coordinates, gene annotations, and MAFs across different breeds. The “Imputation” page showcases the different stages and states of online imputation. Users can download CBP and CMP VCF files from the “Download” page. VCF, Variant Calling Format.

The GCRP offers a user-friendly interface (http://farmrefpanel.com/GCRP/#/), comprising several functional modules (**Figure 5**). First, “VariantDB” allows users to search and visualize detailed data on variants in CBP or CMP, including site position, annotation, and allelic frequency in different populations. Second, the “Imputation” module enables users to run online genotype imputation using three imputation tools: Beagle5.2, Minimac3, and IMPUTE5. Here, users can upload their Variant Calling Format (VCF) files and download the imputation results. Finally, the “Download” module allows users to download VCF files for both CBP and CMP. Users can access these modules by clicking “Download” button to download GCRP VCF files and the corresponding buttons on the “Home” page. The “About” page provides a comprehensive description of the panel’s purpose and components, along with a graphical display of sample geographical distribution, evolutionary distance, and relationships. Finally, users can submit feedback through the email addresses provided on the “Contact Us” page.

## Discussion and perspectives

Genotype imputation is now the standard in genetic research. Comprehensive human genotype imputation panels and platforms, such as 1000 Genomes Project [39] and NyuWa in China [40], have been provided. Universal reference panels have been released for agricultural animals, including cattle [13,41,42], pigs [37,43], and aquatic animals [44]. The Animal-ImputeDB (v2) and AGIDB offer genotype imputation services for various animal species; however, the sample sizes for chickens (*n* = 509 and 1503, respectively) are limited and insufficiently representative [15]. In this study, we established the first public reference panel and genotype imputation web service specifically designed for chicken. To accommodate a variety of application scenarios, we developed two distinct reference panels: the CBP, a specialized resource optimized for commercial breeding purposes, and the CMP, a diverse collection designed to support a broad range of genetic research activities. In the future, the GCRP will serve as a standardized imputation platform in chicken genomics research, enabling large-scale data integration analyses.

The GCRP identified a substantial number of novel variants, including 56% rare mutations (MAF ≤ 0.01), thereby enriching the existing chicken dbSNP database. The results of PCA and phylogenetic analyses on the CBP and CMP indicated that the samples clustered distinctly according to population or origin. Analyses of SNPs, haplotype diversity, and LD decay consistently revealed differences in the genomic backgrounds of commercial and native breeds. Furthermore, we observed that haplotypes between populations exhibited greater differentiation and higher polymorphism compared to SNPs, which is consistent with the historical pattern of relatively isolated evolution among these groups.

Compared with the panels in Animal-SNPAtlas and AGIDB, GCRP (*n* = 11,951) exhibited significant advantages in both SNP array and LCS-based imputation strategies, confirming that GCRP outperforms existing reference panels. This is attributed to the fact that our panel includes a more diverse range of samples, both in terms of quantity and origin. Results from both simulated and real phenotypes demonstrated that post-imputation GWAS generated stronger signals and improved QTL detection power compared to SNP array-based scenarios, confirming that the use of GCRP boosts the statistical power of GWAS. Furthermore, we anticipate that GCRP will play a pivotal role in genomic selection for chickens. Several studies have demonstrated that the use of imputed variants can significantly enhance the accuracy of genomic selection [10,45–47]. GCRP provides a dedicated platform for genotype imputation in commercial chickens, thereby supporting collaborative breeding efforts and the expansion of reference populations.

CBP yielded lower imputation accuracy for domesticated chicken breeds compared to CMP, Animal-SNPAtlas, and AGIDB; however, it performed well when inferring commercial breeds, particularly when using low-density arrays as targets. This highlights the improvement in accuracy achieved by employing large-scale panels, as well as the establishment of specialized panels based on the characteristics of domesticated and commercial chicken breeds. Our results also demonstrate that the imputation strategy based on LCS outperforms those based on low- and high-density arrays, highlighting that sequencing-based approaches are likely to be a superior genotyping strategy for large-scale populations.

Moreover, structural variants (SVs) have garnered increasing attention in recent years; however, SVs are not yet included in the GCRP due to the challenges of detecting SVs using only short-read sequencing data. With the continuous accumulation of data and the increasing availability of long-read platforms, a reference panel incorporating SVs will be introduced in subsequent GCRP versions.

Overall, the objective of this study is to develop a globally applicable genotype imputation platform that facilitates genomic research and breeding applications in chickens. However, there are several challenges that need to be addressed, such as the limited diversity of the current CBP, which includes only five commercial populations, insufficient to fully represent all commercial breeds. Collaborating with additional breeding organizations to incorporate proprietary data into a public database presents significant challenges. However, expanding the dataset and establishing further collaborations to continuously update the CBP and CMP will remain a key focus of our future work. For example, GCRP (v2) will incorporate African local breeds and commercial laying breeds, offering a comprehensive panel for chicken genotype imputation. We anticipate that this extensive collection will serve as a valuable genetic resource for studying the evolution and enhancement of chickens and other avian species. Moreover, integrating GCRP with more advanced genomic or multi-omics research presents a significant future challenge. To address this, our project will collaborate with the Functional Annotation of Animal Genomes (FAANG) project [48], and gradually integrate the GCRP with regulatory elements atlas [49] and ChickenGTEx [50]. This integration can then be applied to meta and colocalization analyses [51,52], facilitating a more comprehensive analysis of gene regulatory mechanisms, while also contributing to the development of more accurate artificial intelligence algorithms [53,54].

## Ethical statement

All animal procedures in this study were conducted in accordance with the guidelines of the Institutional Animal Care and Use Committee (IACUC) of China Agricultural University (Approval No. SKLAB-2014-06-07).

## Code availability

The code used in the study is available from the GitHub repository (https://github.com/desindygogo/GCRP_pipeline). The code has also been submitted to BioCode at the National Genomics Data Center (NGDC), China National Center for Bioinformation (CNCB) (BioCode: BT007714), which is publicly accessible at https://ngdc.cncb.ac.cn/biocode/tool/BT007714.

## Supplementary Material

qzaf032_Supplementary_Data

## Data Availability

The raw data for 2545 chickens in the CBP and 64 high-depth sequencing samples used for validation have been deposited in the Genome Sequence Archive [55] at the NGDC, CNCB (GSA: CRA022364 and CRA022446, respectively), and are publicly accessible at https://ngdc.cncb.ac.cn/gsa. The remaining data are available upon scientific request from the corresponding author. GCRP has been submitted to Database Commons [56] at the NGDC, CNCB, which is publicly accessible at https://ngdc.cncb.ac.cn/databasecommons/database/id/9708.

## CRediT author statement

**Di Zhu:** Writing − original draft, Data curation, Formal analysis, Methodology, Visualization, Writing − review & editing, Software. **Yuzhan Wang:** Formal analysis, Visualization. **Hao Qu:** Data curation, Resources. **Chungang Feng:** Writing − review & editing, Methodology. **Hui Zhang:** Writing − review & editing, Data curation. **Zheya Sheng:** Data curation. **Yunliang Jiang:** Writing − review & editing. **Qinghua Nie:** Writing − review & editing. **Suqiao Chu:** Data curation, Resources. **Dingming Shu:** Writing − review & editing, Resources. **Ziqin Jiang:** Data curation, Resources. **Dexiang Zhang:** Data curation, Resources. **Lingzhao Fang:** Writing − review & editing. **Hui Li:** Writing − review & editing, Data curation. **Zhenqiang Xu:** Data curation, Resources. **Yiqiang Zhao:** Supervision, Writing − review & editing, Data curation. **Yuzhe Wang:** Conceptualization, Project administration, Supervision, Writing − review & editing, Methodology, Funding acquisition. **Xiaoxiang Hu:** Funding acquisition, Supervision, Writing − review & editing. All authors have read and approved the final manuscript.

## ORCID

0000-0002-9362-3834 (Di Zhu)

0000-0003-4463-4075 (Yuzhan Wang)

0000-0001-6561-4271 (Hao Qu)

0000-0002-7031-4211 (Chungang Feng)

0000-0002-6331-6876 (Hui Zhang)

0000-0002-4487-2682 (Zheya Sheng)

0000-0002-2914-6818 (Yunliang Jiang)

0000-0003-3934-3820 (Qinghua Nie)

0009-0005-3176-0568 (Suqiao Chu)

0000-0002-0109-3013 (Dingming Shu)

0009-0005-9538-7539 (Ziqin Jiang)

0009-0006-1574-0994 (Dexiang Zhang)

0000-0003-1103-3679 (Lingzhao Fang)

0000-0001-6282-5559 (Hui Li)

0009-0007-8633-0041 (Zhenqiang Xu)

0000-0002-0076-3476 (Yiqiang Zhao)

0000-0003-3474-7957 (Yuzhe Wang)

0000-0001-7045-0283 (Xiaoxiang Hu)
