# Supplementary material for: GCRP: Integrated Global Chicken Reference Panel from 11,951 Chicken Genomes
Source: Genomics Proteomics Bioinformatics. 2025 Apr 15;23(3):qzaf032. doi: 10.1093/gpbjnl/qzaf032 (PMC12458076; doi:10.1093/gpbjnl/qzaf032)
Supplement: qzaf032_Supplementary_Data [file qzaf032_supplementary_data.zip › Figure S3.pdf]

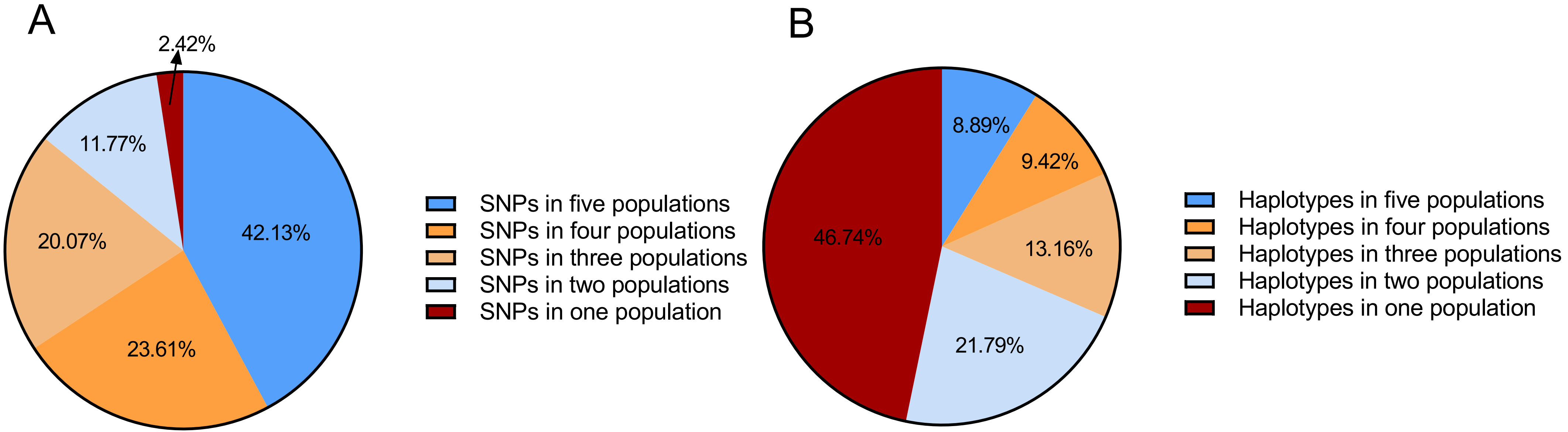

A
2.42%
11.77%
20.07%
42.13%
23.61%
SNPs in five populations
SNPs in four populations
SNPs in three populations
SNPs in two populations
SNPs in one population
B
8.89%
9.42%
13.16%
46.74%
21.79%
Haplotypes in five populations
Haplotypes in four populations
Haplotypes in three populations
Haplotypes in two populations
Haplotypes in one population
