# Supplementary material for: GCRP: Integrated Global Chicken Reference Panel from 11,951 Chicken Genomes
Source: Genomics Proteomics Bioinformatics. 2025 Apr 15;23(3):qzaf032. doi: 10.1093/gpbjnl/qzaf032 (PMC12458076; doi:10.1093/gpbjnl/qzaf032)
Supplement: qzaf032_Supplementary_Data [file qzaf032_supplementary_data.zip › Supplementary material captions.docx]

**Supplementary materials**

**Figure S1 The STITCH parameter K gradient test in CBP**

The X-axis represents the K values set in the range of 5 to 40, and the Y-axis represents the genotype concordance between CBP and 64 gold standard datasets. The test was conducted using chromosome 6. STITCH, Sequencing To Imputation Through Constructing Haplotypes.

**Figure S2 Details of variations in GCRP**

**A.** Venn diagram depicting the overlap of SNP among dbSNP, CBP, and CMP. **B.** Venn diagram depicting the overlap of indel among dbSNP and CMP. **C.** Histogram showing the distribution of variant MAFs in CMP. **D.** Histogram showing the distribution of variant MAFs in CBP. **E.** The left Y-axis represents GC between CBP and 64 high-depth sequencing validation samples, the right Y-axis corresponds to the sequencing depth of the validation samples. **F.** Allele dosage r² between the CBP and 64 high-depth sequencing validation samples. The X-axis represents MAF bins (1%). indel, insertion and deletion; dbSNP Single Nucleotide Polymorphism Database.

**Figure S3 The sharing of variations and haplotypes within CBP**

**A.** Proportion of shared SNPs in CBP across five populations. **B.** Proportion of shared haplotypes in CBP across five populations.

**Figure S4 The dosage r^2^ under different imputation scenarios**

Square of the correlation between the imputed allele dosage and the true allele dosage for each panel and imputation strategy combination for 205 test samples (**A**), 66 commercial WPR samples (**B**), and 139 non-commercial samples (**C**).

**Figure S5 The computation time cost by different genotype imputation software**

All test used chromosome 6 under the same conditions (5 central processing units and no memory limitations) for 205 test samples.

**Figure S6 The genotype concordance under different imputation scenarios**

The genotype concordance of imputed versus observed genotypes using different software and three reference panels targeted to Illumina 60 k array, Affymetrix 600 k, and low-coverage sequence for 66 commercial WPR samples (**A–C**) and 139 non-commercial samples (**D–F**)**.**

**Figure S7 Comparison of imputation accuracy between SNPs and indels using CMP**

The X-axis represents different imputation scenarios.

**Figure S8 The imputation allele dosage r^2^ with different MAF bins using QUILT**

**Table S1 Detail information for** **comprehensive mix panel samples**

**Table S2 Detail information for** **commercial breed panel populations**

**Table S3 Detail information for 205 test samples for imputation assessment**
