# Supplementary figures and images for: GCRP: Integrated Global Chicken Reference Panel from 11,951 Chicken Genomes
Source: Genomics Proteomics Bioinformatics. 2025 Apr 15;23(3):qzaf032. doi: 10.1093/gpbjnl/qzaf032 (PMC12458076; doi:10.1093/gpbjnl/qzaf032)

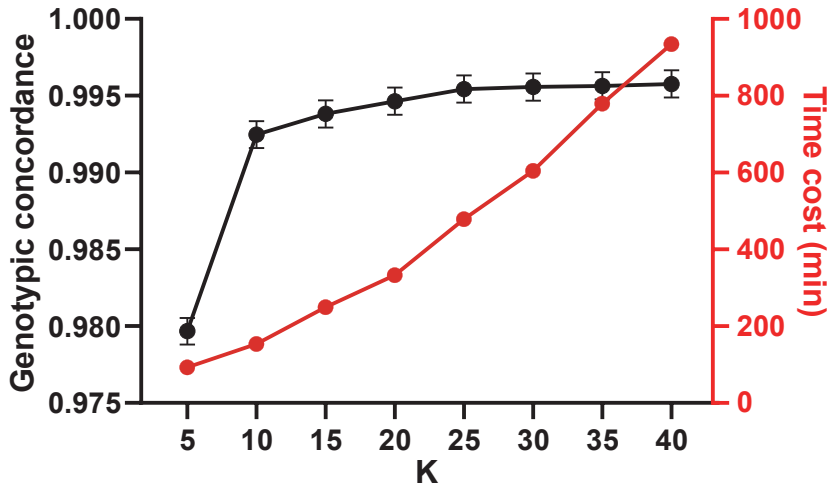

Supplement: qzaf032_Supplementary_Data [file qzaf032_supplementary_data.zip › Figure S1.pdf]

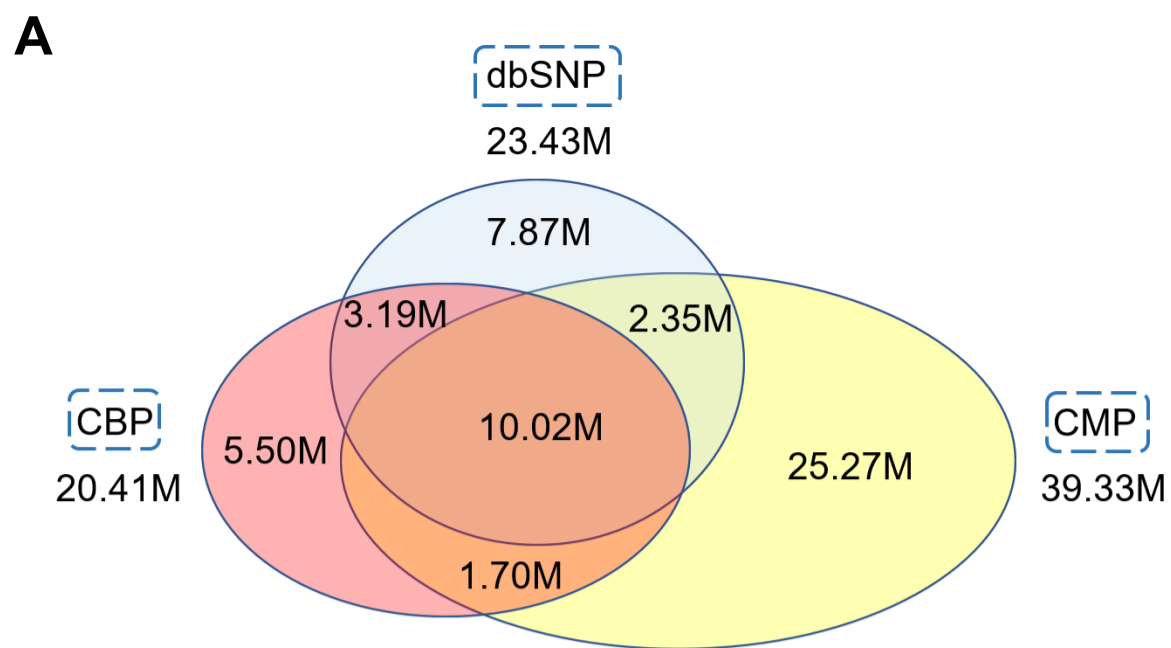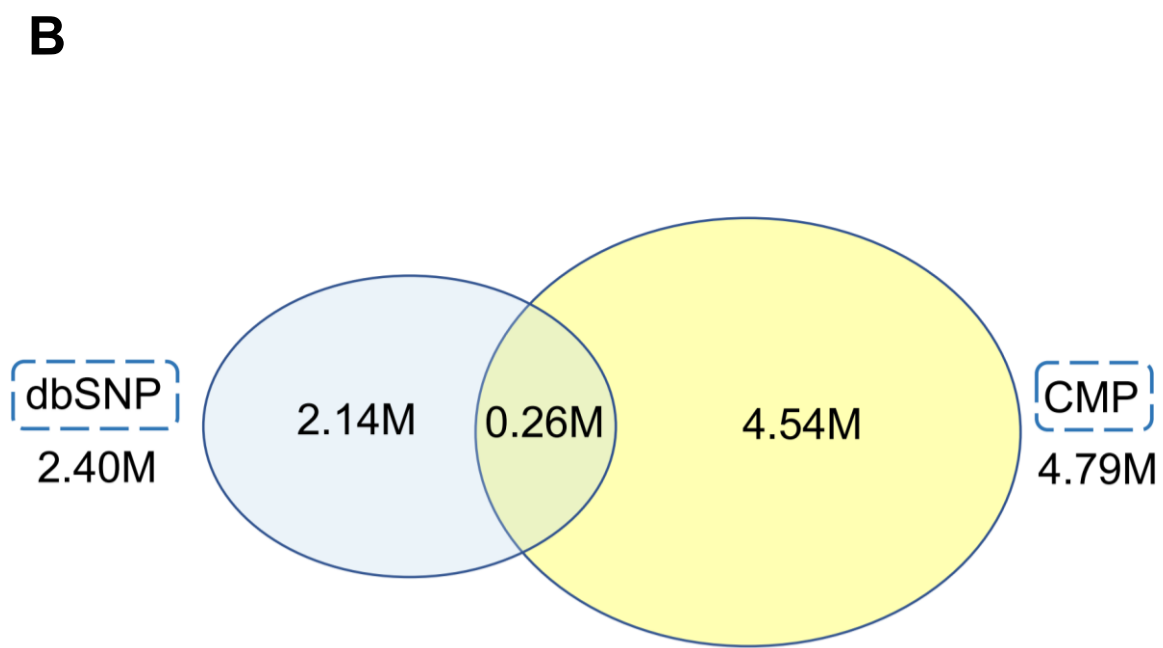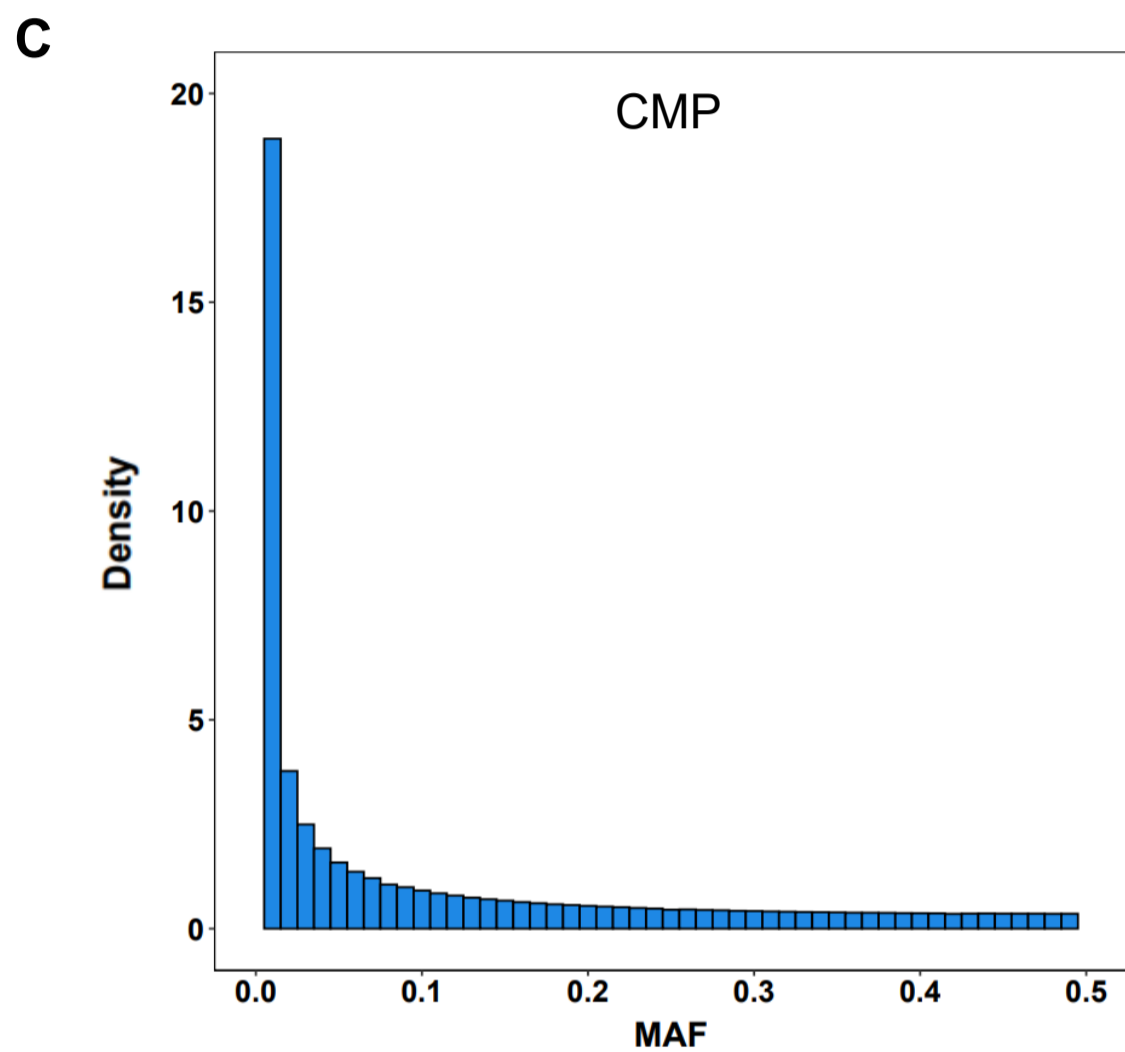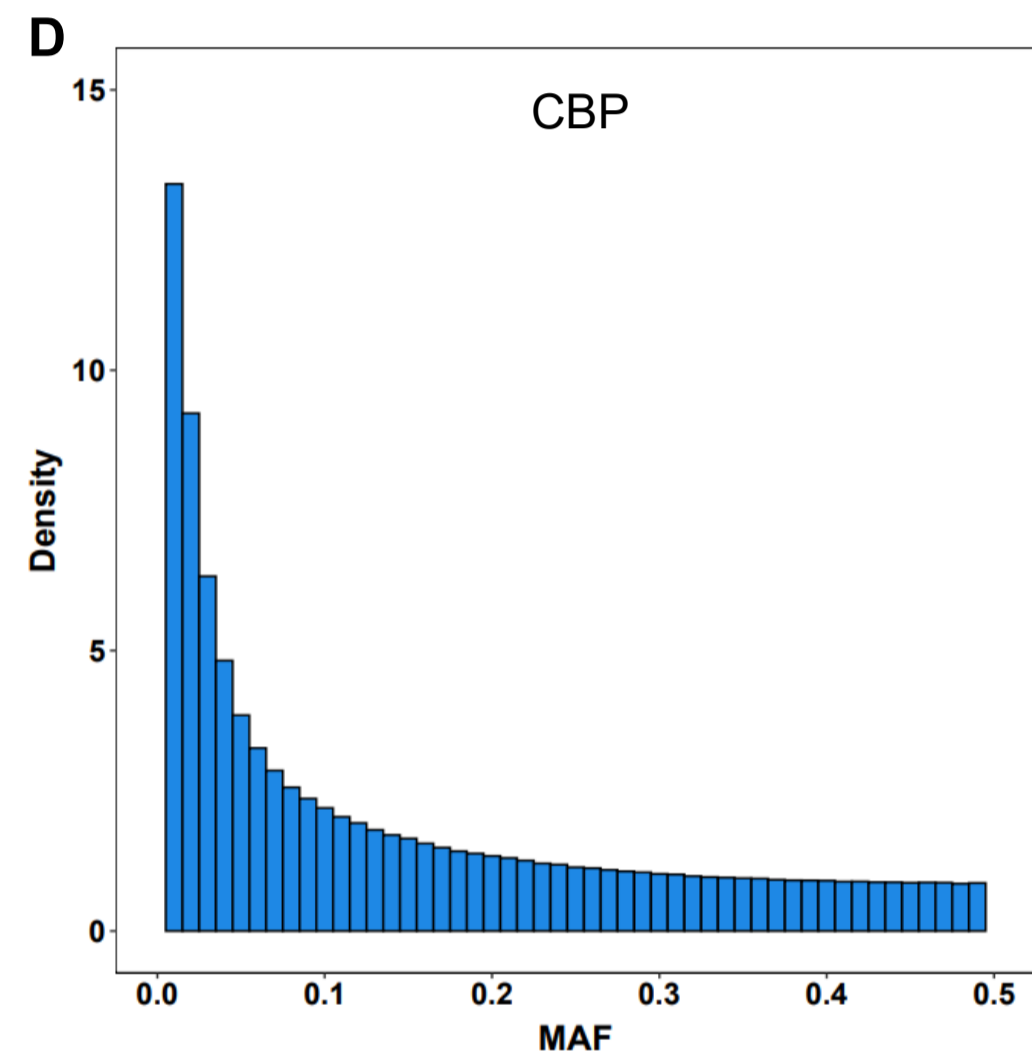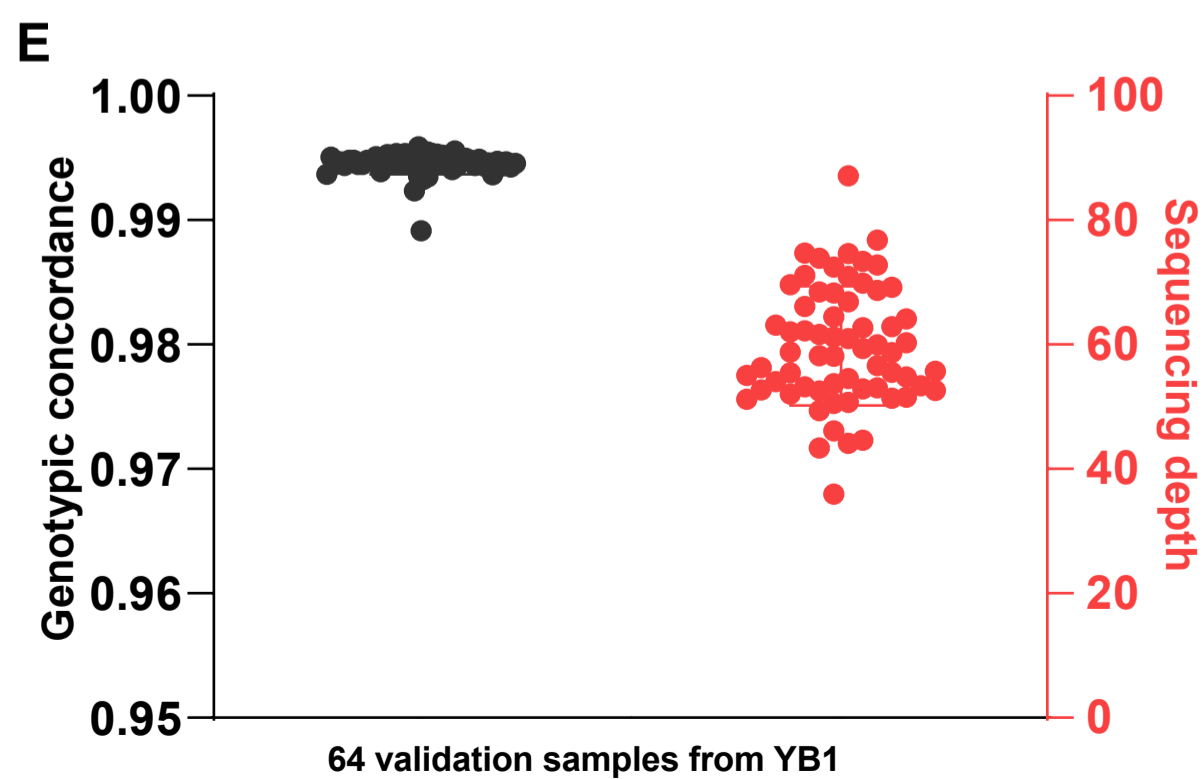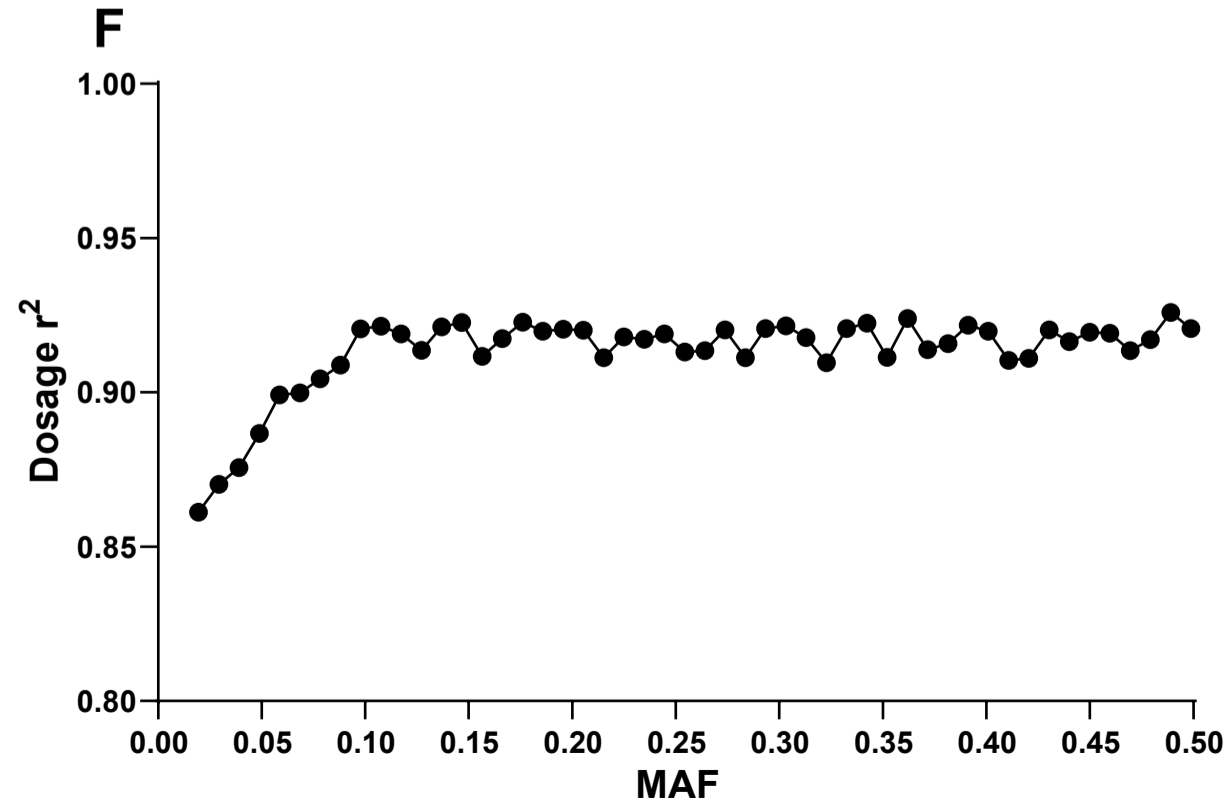

Supplement: qzaf032_Supplementary_Data [file qzaf032_supplementary_data.zip › Figure S2.pdf]

**A****205 test samples**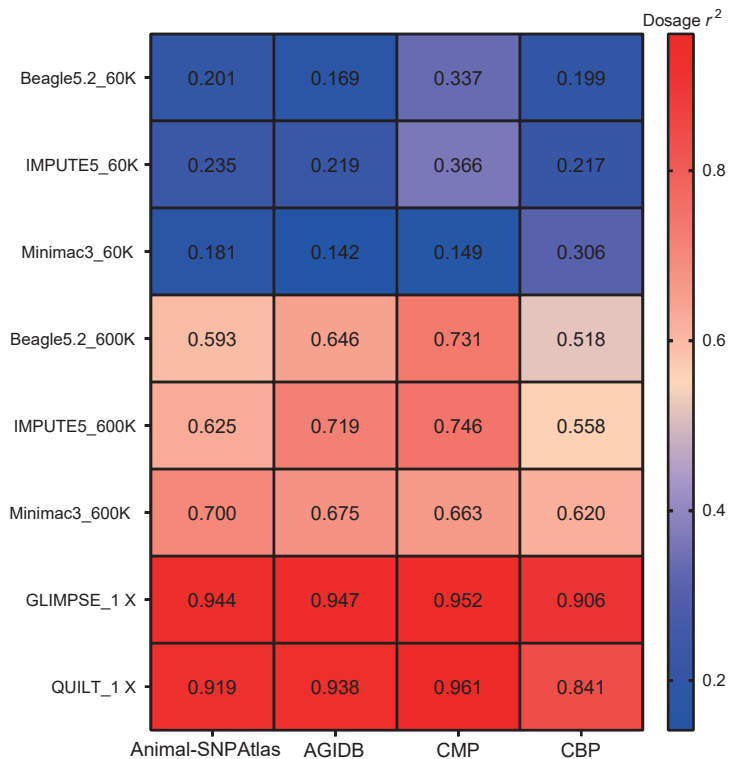**B****66 Commercial samples**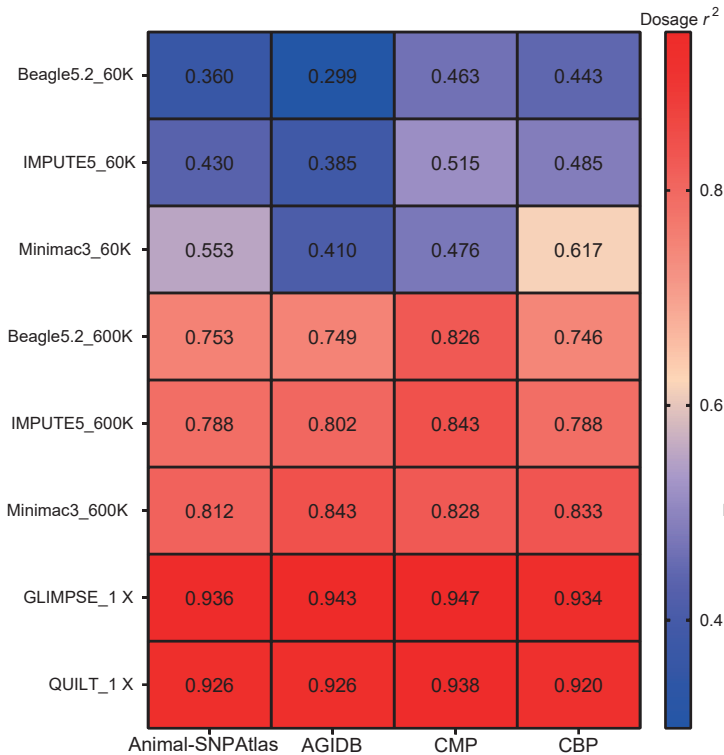**C****139 Non-commercial samples**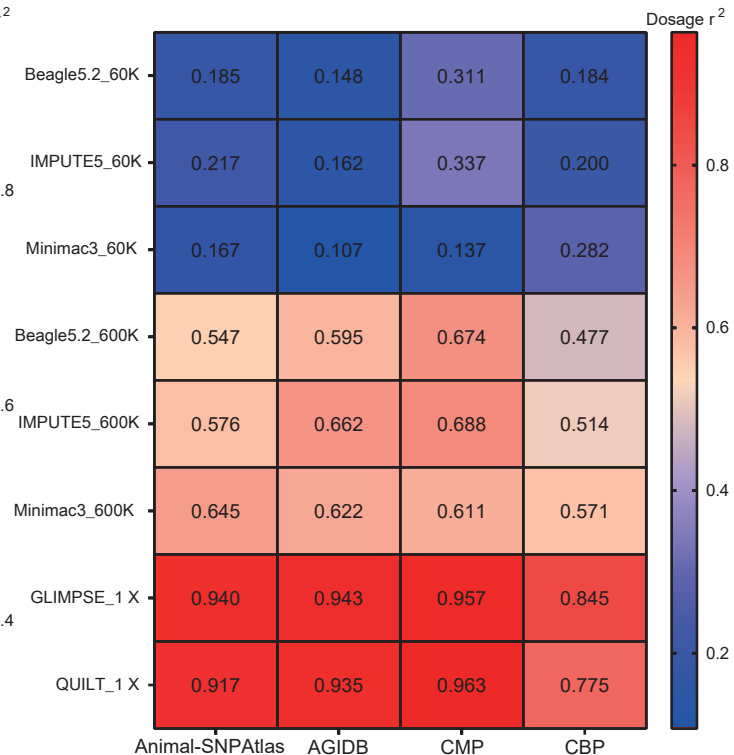

Supplement: qzaf032_Supplementary_Data [file qzaf032_supplementary_data.zip › Figure S4.pdf]

**Imputation software**

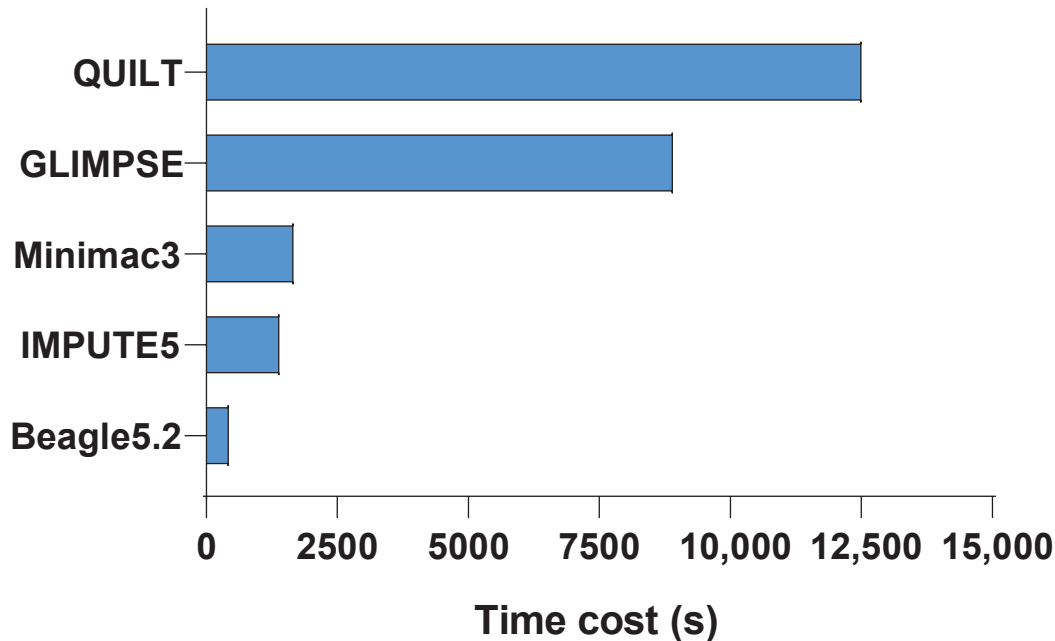

Supplement: qzaf032_Supplementary_Data [file qzaf032_supplementary_data.zip › Figure S5.pdf]

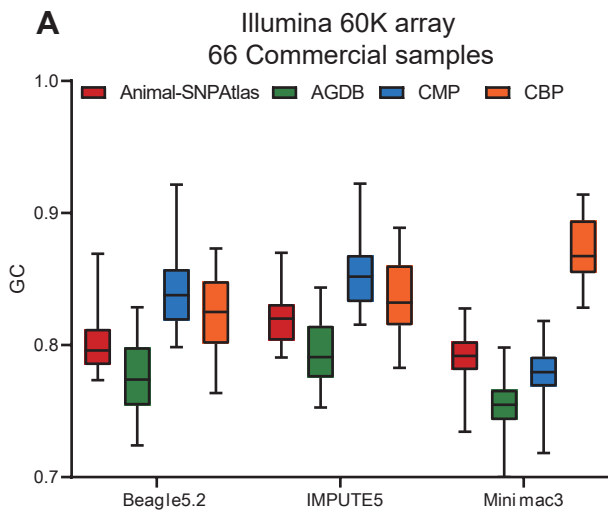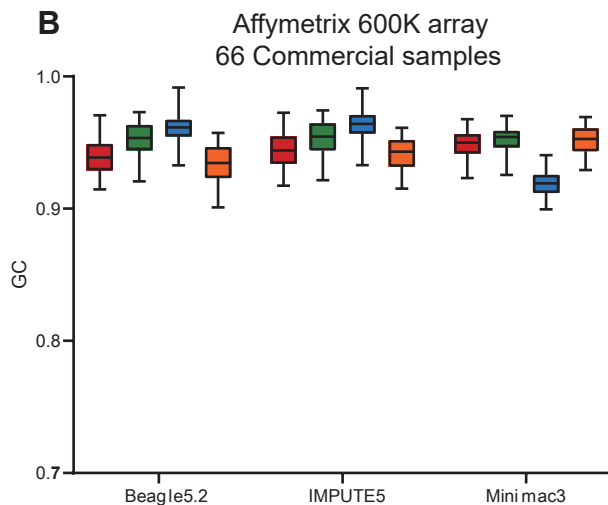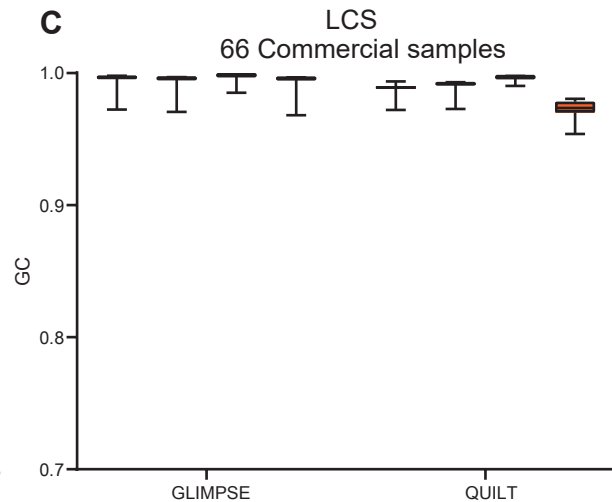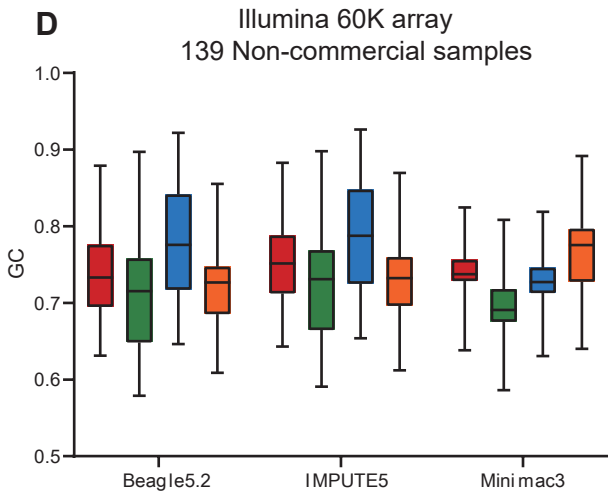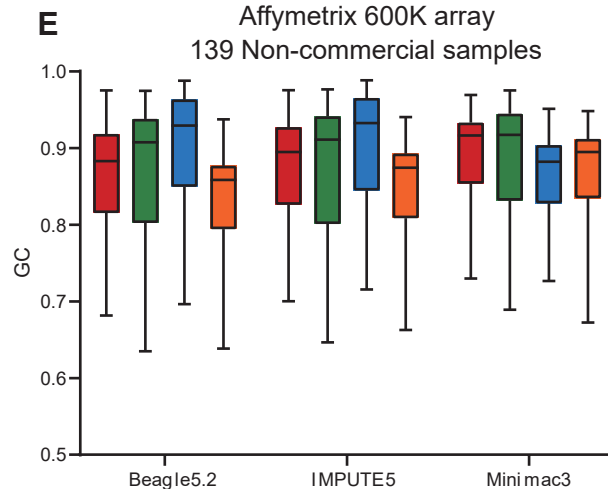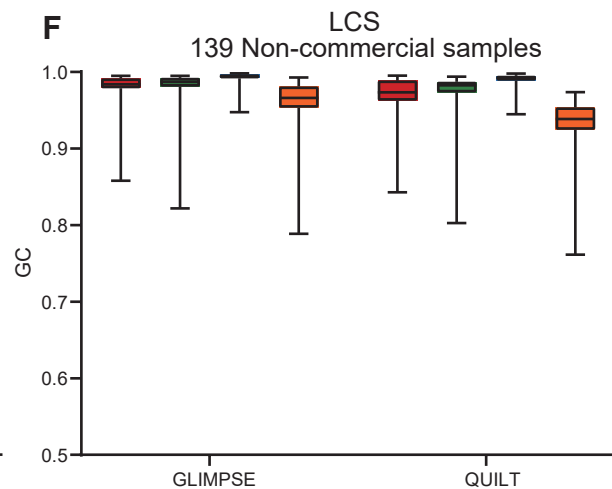

Supplement: qzaf032_Supplementary_Data [file qzaf032_supplementary_data.zip › Figure S6.pdf]

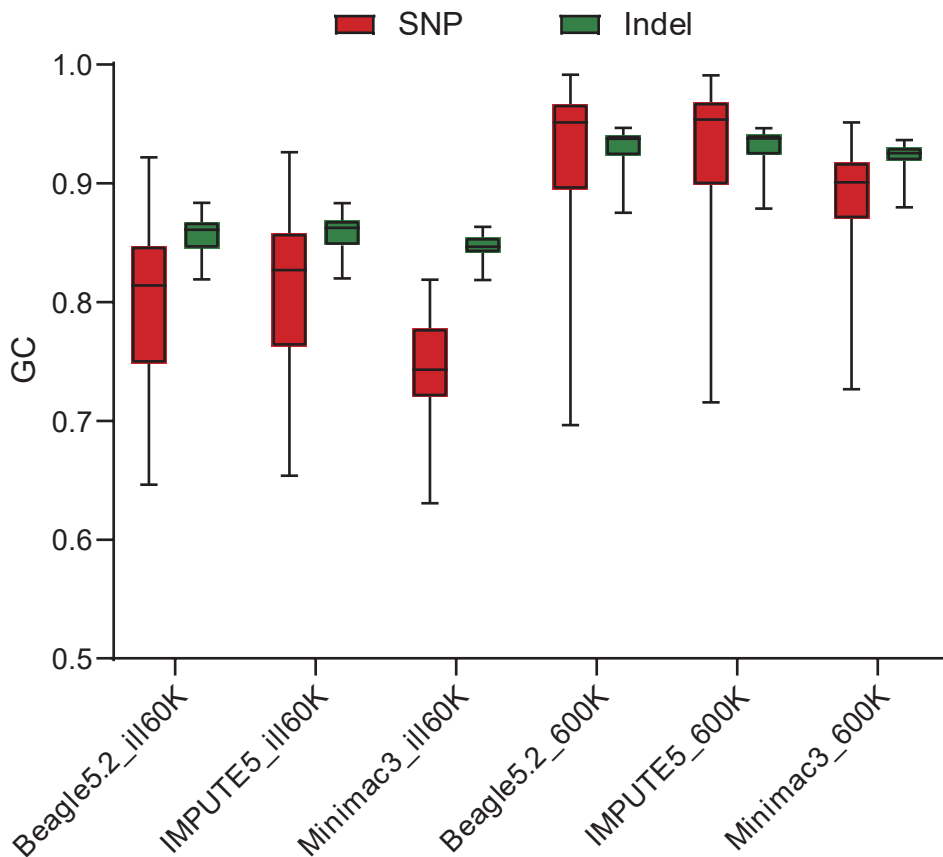

Supplement: qzaf032_Supplementary_Data [file qzaf032_supplementary_data.zip › Figure S7.pdf]

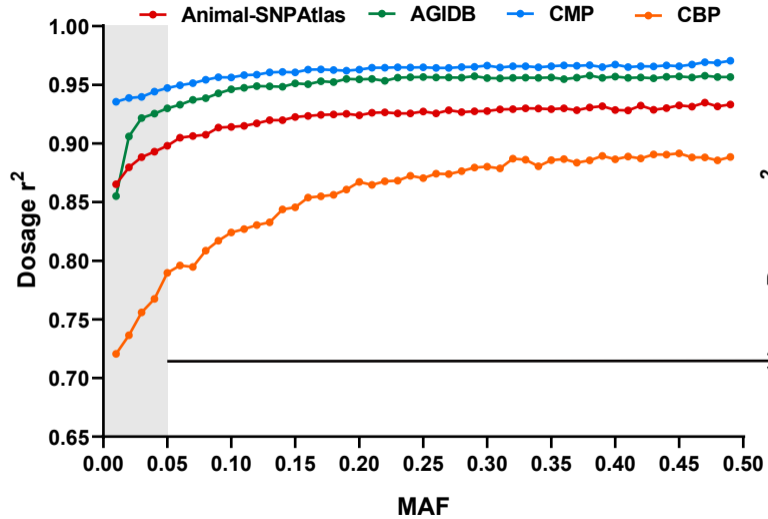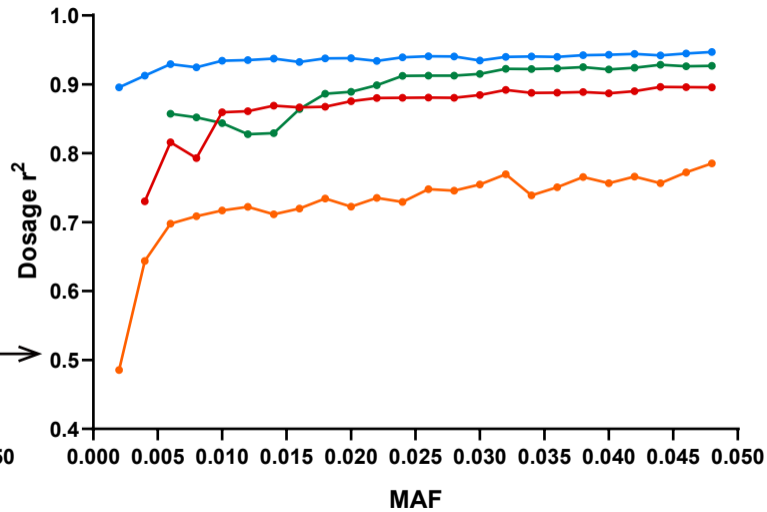

Supplement: qzaf032_Supplementary_Data [file qzaf032_supplementary_data.zip › Figure S8.pdf]
